# Anovestibular Fistula in An Infant with Retroviral Infection − Case Report

**DOI:** 10.34763/devperiodmed.20172102.98100

**Published:** 2017-08-11

**Authors:** Deepa Makhija, Hemanshi Shah, Charu Tiwari, Jayesh Desale

**Affiliations:** 1 Dept of Paediatric Surgery, TNMC & BYL Nair Hospital, Mumbai Central, Mumbai Maharashtra. India. Pin:400008

**Keywords:** retroviral infection, anovestibular fistula, congenital, acquired

## Abstract

A vestibular fistula with a normal anus is a rare subtype of anorectal malformation seen more often in East Asia and India. Though mostly congenital, some authors have suggested acquired etiologies for this condition. Infants with retroviral infection have been reported to develop acquired rectovestibular fistulas. We report a case of an infant anovestibular fistula in a patient with retroviral infection.

## Introduction

A vestibular fistula with a normal anus is a rare subtype of anorectal malformation (ARM). It represents only 3% of anorectal malformations worldwide [1]. The highest incidence is in East Asia and India, where it has been reported to represent up to 12.5% of anorectal malformations [1]. Although first described in 1960, there remains no consensus on the etiopathogenesis and optimal management of this condition [1]. Surgical repairs described in the literature range from simple excision and closure of the fistula [1] to a complete anorectoplasty with division and repair of sphincters [1]. Acquired etiology for this condition has also been proposed [2]. We describe a case of an anovestibular fistula in a child diagnosed as retroviral positive.

## Case summary

**Fig. 1:**
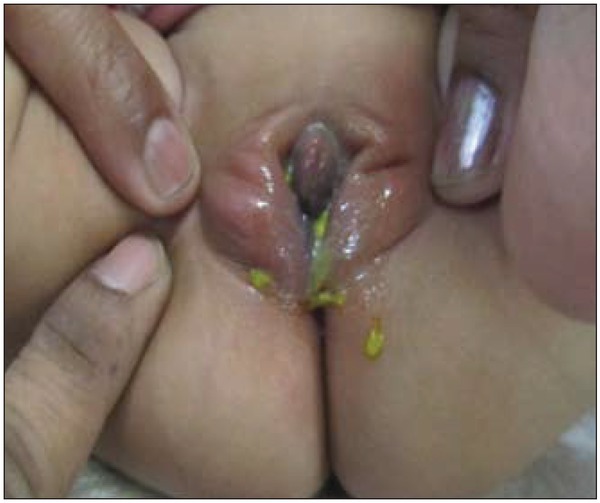
Local examination of the perineum showing stools from the vestibule, Surrounding inflammation and normal anus.

**Fig. 2:**
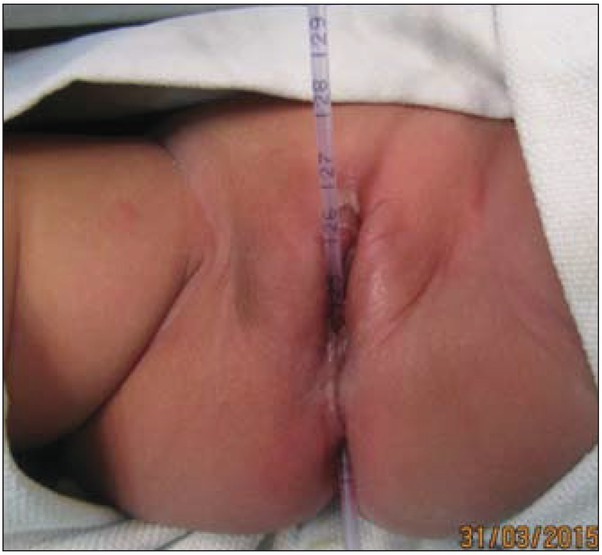
Intraoperative image with an Infant Feeding Tube through the fistula.

**Fig. 3:**
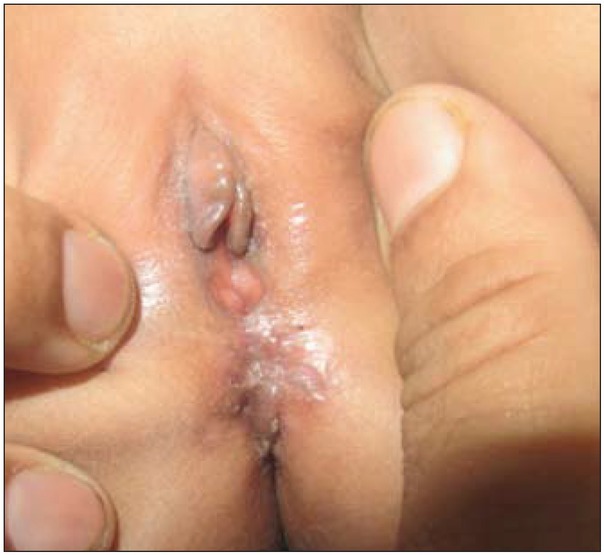
Post-operative photograph of the perineum.

A 4 month-old girl was admitted with a history of passing stools from the vestibule for a month and a half. She had a history of passing stools per anum since birth. There was also a history of a swelling in the right inguinal region for one month and of diarrhea for one week. On examination, a normal anal opening was present at the normal site. There was redness in the perineum and stools were seen coming from the vestibule (fig. 1). Bilateral inguinal lymphadenopathy was present. On investigation, the patient had an elevated white blood count. Ultrasonography showed bilateral inguinal lymphadenopathy. The mother and child were found to be seropositive. The patient was started on conservative management by maintaining local hygiene, sitz bath, oral antiobiotics and anal dilatations. Anti-retroviral therapy was started for the mother and child. The fistula persisted after 6 weeks of conservative management. The patient was posted for surgery. At surgery, the fistulous tract could be palpated anteriorly and tract excision with anoplasty was done (fig. 2). The patient has been passing stools from the normal orifice post surgery (fig. 3).

## Discussion

Vestibular fistula with a normal anus is a rare condition and has been variously described in the literature as H-type fistula, perineal canal, N-type fistula and double termination of alimentary tract [3]. The H-type of fistula has been included in the “Rare/regional variant Anorectal Malformation” in the Krickenbeck classi! cation and refers to patients who have a congenital rectourogenital connection and a normally or ectopically placed external anal opening [3].

The embryologic basis for this malformation is not well established and multiple mechanisms have been proposed [4]. The embryologic etiology may not be the same for males and females [4]. Stephen [2, 5] has proposed impaired alignment of the cranial and caudal septae dividing the internal cloaca as the etiology of the H-type fistula. De Vries and Friedland [2] described the H-type fistula in males as the persistence of the cloacal duct of Reichel, which links anterior and posterior compartments of the cloaca [3, 4]. The most widely accepted theory is the interruption of the dorsal part of the cloacal membrane by an isolated defect [2].

Although considered congenital, acquired etiology has also been suggested for the anovestibular fistula [2]. Acquired fistulas have been found associated with vulvar abscess [2], preceding an episode of diarrhoea [6] and retroviral disease in the mother [3]. Acquired fistula has been proposed as an early manifestation of retroviral disease in infants. The pathogenesis of these acquired fistulas as suggested by Tushida et al involves failure of migration of urorectal and uroanal septum with excessive posterior fusion of the genital folds that might cause a patent or a partly patent anorectovestibular fistula despite a normal anal opening [2]. In cases of inflammation in a partly patent fistula, abnormal opening(s) may form at the vestibule [2]. The presence of multiple openings, associated local in$ammation and lateral position of the vestibular opening suggest an acquired disease.

Retroviral infection is a multi-systemic infection with increased incidence in developing countries [7]. Acquired vestibular fistulas, though rare, have been reported in children with retroviral infections [7]. Routine exclusive breastfeeding has been postulated to result in the transmission of a retroviral infection from the mother to the infant [7]. The acquired immunode! ciency syndrome manifests as severe perineal inflammation and is compounded by infection by various organisms [7]. Continuous faecal contamination perpetuates this infection and inflammation, thereby keeping the fistula patent [7]. Such cases with retroviral infection and diarrhoea in infancy presenting with fistulas have been reported from the African subcontinent [7].

The diagnosis of this variant of vestibular fistula is usually missed in the neonatal period, because of the presence of a normal anal opening. Patients usually present in infancy with complaints of passing stools through the vestibule, as well as from the normally positioned anus. Other symptoms include stool in the urine in males, recurrent perineal infections, multiple episodes of urinary tract infections and straining during defecation [2, 4].

The management of these fistulas is individualized. The first line of management is always conservative. Treatment involves local hygiene, antibiotics and anal dilatations. Routine antenatal retroviral screening and appropriate antiretroviral treatment in positive cases is necessary in order to reduce the risk of transmission of this infection to babies. In those patients for whom the conservative trail fails, surgery is considered. Multiple approaches and surgical procedures alone or in combination have been described in the literature including vestibulo-anal pull through, anterior rectal wall pull through, limited PSARP, endorectal pull through and direct excision [2]. The procedure may or may not be combined with a colostomy [2]. A diverting colostomy has not been shown to help in the closure of the fistula or to prevent recurrences [2]. Recurrence and wound dehiscence are the two frequently reported complications [8]. Prognosis is good as far as anal continence is concerned as the anal sphincter complex development is normal [4].
